# Rauchen und Nikotinkonsum, erhitzte Tabakprodukte, psychische und sozioökonomische Aspekte von Dependenz, Alkohol, Infektionen, Impfen und Diabetes mellitus (Update 2026)

**DOI:** 10.1007/s00508-025-02680-x

**Published:** 2026-04-30

**Authors:** Helmut Brath, Simone Huber, Verena Parzer, Martin Riesenhuber, Christian Tatschl, Peter Fasching, Martin Clodi

**Affiliations:** 1 Sigmund Freud Privatuniversität Medizin, Campus Prater, Wien, Österreich; 2 Diabetes- und Fettstoffwechselambulanz, Mein Gesundheitszentrum Favoriten, Wien, Österreich; 3 1. Medizinische Abteilung für Endokrinologie, Diabetologie und Stoffwechsel, Klinik Landstraße, Wien, Österreich; 4 Zentrum für Innere Medizin ZIM9, Wien, Österreich; 5 Psychotherapeutische Praxis Sinn 360, Wien, Österreich; 6 5. Medizinische Abteilung mit Endokrinologie, Rheumatologie und Akutgeriatrie, Klinik Ottakring, Wien, Österreich; 7 Abteilung für Innere Medizin, Krankenhaus der Barmherzigen Brüder, Linz, Österreich; 8 Clinical Research Institute for Cardiovascular and Metabolic Diseases, Medizinische Fakultät, Johannes Kepler Universität Linz, Linz, Österreich

**Keywords:** Rauchen, Alternative Tabak- und Nikotinprodukte, Alkohol, Infektionen und Impfungen, Diabetes mellitus, Smoking, Alternative tobacco and nicotine products, Alcohol, Infections and vaccinations, Diabetes mellitus

## Abstract

Rauchen und Passivrauchen steigern signifikant die Diabetesinzidenz sowie das Risiko für Spätschäden. Ein vollständiger Rauch- und Nikotinstopp kann zwar zu Gewichtszunahme und erhöhtem Diabetesrisiko führen, senkt jedoch die kardiovaskuläre und Gesamtmortalität. Die Basisdiagnostik (Fagerström-Test, exhalatorisches CO) ist essenziell für eine erfolgreiche Raucherentwöhnung. Evidenzbasierte medikamentöse Optionen umfassen Vareniclin, Nikotinersatztherapie, Bupropion und Cytisin; GLP-1-Rezeptoragonisten können adjuvant zur Gewichtskontrolle eingesetzt werden. Weder erhitzte Tabakprodukte, E‑Zigaretten noch rauchfreie Nikotinprodukte (Nikotinbeutel) sind harmlose Alternativen – sie sind ebenfalls mit erhöhtem Diabetesrisiko, Morbidität und Mortalität assoziiert. Sozioökonomische und psychische Faktoren beeinflussen Suchtverhalten (Rauchen, Nikotin, Alkohol) maßgeblich. Moderater Alkoholkonsum scheint das Diabetes- und kardiovaskuläre Risiko zu senken, doch Selektionsbias und Untererfassung relativieren diesen Effekt. Dosisabhängig überwiegen jedoch die Risiken durch Krebs, Lebererkrankungen und Infektionen. Infektionen treten bei Diabetes häufiger auf, verlaufen oft schwerer und atypisch. Bei unklarer Hyperglykämie ist immer an eine Infektion zu denken. Impfungen sind für Diabetespatient:innen von besonders hoher gesundheitlicher Relevanz.

## Abstract (en)

Smoking and passive smoking significantly increase both the incidence of diabetes and the risk of late complications. While complete smoking and nicotine cessation may lead to weight gain and an increased risk of diabetes, it nevertheless reduces cardiovascular and all-cause mortality. The basic diagnostic assessment (Fagerström test, exhaled carbon monoxide) is essential for successful smoking cessation. Evidence-based pharmacological options include varenicline, nicotine replacement therapy, bupropion and cytisine; glucagon-like peptide 1 (GLP-1) receptor agonists may be used as adjunctive treatment for weight control following smoking cessation. Neither heated tobacco products, e‑cigarettes nor smokeless nicotine products (nicotine pouches) are harmless alternatives, they are also associated with an increased risk of diabetes, morbidity, and mortality. Socioeconomic and psychological factors play a significant role in addictive behavior, including smoking, nicotine use and alcohol consumption. Moderate alcohol consumption appears to reduce the risk of diabetes and cardiovascular disease but selection bias and underreporting likely overestimate this effect. Dose-dependent risks, particularly through cancer, liver disease and infections predominate. Infections occur more frequently in individuals with diabetes, often with greater severity and atypical presentation. Unexplained hyperglycemia should always prompt consideration of an infection. Vaccinations are of even greater health relevance for patients with diabetes than for those without.

## 1. Rauchen und Nikotinkonsum, erhitzte Tabakprodukte

### Rauchen

Rauchen ist eine der wichtigsten weltweiten Ursachen für erhöhte Morbidität und Mortalität. Über 10 % der weltweiten Mortalität sind rauchbedingt [1], ca. 1 % der weltweiten Mortalität ist auf Passivrauchen zurückzuführen [2]. Bereits eine Zigarette pro Tag erhöht das Risiko für koronare Herzkrankheit um 48 % (Männer) bzw. um 57 % (Frauen) und das Insultrisiko um 25 % (Männer) bzw. 31 % (Frauen) [3]. Im Schnitt geht die Rauchprävalenz in Österreich und weltweit zurück [4].

Aktivrauchen erhöht bereits bei Menschen ohne Diabetes die durchschnittlichen Blutzuckerwerte [5] und die Inzidenz von Typ-2-Diabetes um 37 bis über 100 % [6–8]. Passivrauchen steigert die Diabetesinzidenz um mehr als 30 % [9]. Rauchen erhöht die Wahrscheinlichkeit für diabetische Spätschäden, z. B. der Nephropathie [10]. Aktivrauchen erhöht das relative Risiko von rauchenden vs. nicht rauchenden Patient:innen mit Diabetes auf 1,55 (Gesamtmortalität), 1,49 (kardiovaskuläre Mortalität), 1,51 (koronare Herzkrankheit), 1,54 (Insult), 2,15 (paVK) und 1,43 (Herzinsuffizienz). Patient:innen, die das Rauchen aufgaben, hatten zwar weiterhin gegenüber Nichtrauchern erhöhte Risiken bezüglich Gesamtmortalität und kardiovaskulären Erkrankungen, diese waren jedoch deutlich geringer als die Risiken jener, die weiterhin rauchten [11]. Daten aus dem schwedischen Diabetesregister zeigen, dass Rauchen der wichtigste singuläre Risikofaktor zur Prädiktion der Gesamtmortalität bei Patient:innen mit Diabetes ist [12].

Eine Zusammenfassung der Daten findet sich z. B. in einem rezenten Positionspapier der Société Francophone de Tabacologie und der Société Francophone du Diabète [13]. Ursächlich werden eine erhöhte Insulinresistenz, eine vermehrte Akkumulation viszeralen Fettes, erhöhte Spiegel von Cortisol und Schilddrüsenhormonen, erhöhter Sympathikustonus, erhöhte systemische Inflammation sowie eine Reduktion der Insulinfreisetzung und eine reduzierte Betazellmasse diskutiert [14].

Neueste Daten zeigen zudem, dass die antidiabetische Therapieeffizienz bei Rauchern geringer ist als bei Nichtrauchern: Der HbA_1c_ sank bei 757 untersuchten rauchenden Männern mit Diabetes um 0,33 % geringer ab als bei nicht rauchenden. Bei einem BMI < 25 kg/m^2^ war die Differenz sogar 0,74 % [15].

### Gewichtszunahme nach Rauch- und Nikotinstopp und diabetesspezifische Aspekte

Ein Rauch- und Nikotinstopp kann von einer unerwünschten Gewichtszunahme, vermutlich durch Appetitzunahme und Reduktion des Grundumsatzes, begleitet sein. Laut einer Cochrane-Metaanalyse gelingt es zwar 16 % der Ex-Rauchenden, gleichzeitig mit dem Rauchstopp abzunehmen, 13 % nehmen aber mehr als 10 kg zu [15]. Dies führte laut einer Metaanalyse dazu, dass der Rauchstopp mit einer mittelfristigen Erhöhung des Risikos für Typ-2-Diabetes (Höhepunkt nach 5 bis 7 Jahren, langfristig aber Absenken unter das Risiko von Rauchenden) einherging; diese Risikoerhöhung war dem Ausmaß der Gewichtszunahme direkt proportional. Trotz des erhöhten Diabetesrisikos hatten aber jene, die das Rauchen aufgegeben hatten, ein signifikant geringeres Risiko für kardiovaskulären Tod und nahezu eine Halbierung der Gesamtmortalität [16]. Gewichtszunahme und passager erhöhtes Diabetesrisiko reduzieren den Nutzen des Rauchstopps somit nicht.

Zigarettenrauchen ist ein substanzieller Risikofaktor für bakterielle und virale Infektionen [17], Gleiches gilt für Diabetes [18]. Leider rauchen Menschen mit chronischen Infektionskrankheiten, wie z. B. HIV, häufiger als die Allgemeinbevölkerung [19]. Ein Rauch- und Nikotinstopp sollte deswegen eine unbedingte Priorität bei rauchenden Personen mit Komplikationen sein [17].

### Diagnostik und Therapie

Zur Basisdiagnostik des Rauchverhaltens sollte der Grad der körperlichen Abhängigkeit mittels des Fagerström-Tests oder Heaviness of Smoking-Index erhoben werden [20]. Bei höherem Abhängigkeitsgrad empfiehlt sich die medikamentöse Unterstützung des Rauch- bzw. Nikotinstopps. Eine mit einem einfachen Handmessgerät durchführbare Messung des exhalatorischen CO-Wertes kann falsche anamnestische Angaben aufdecken und den Erfolg einer Raucherentwöhnung dokumentieren. Ebenso bietet sich der Cotinin-Urintest an.

Ein Überblick über die Methoden der Raucherentwöhnung wurde in der Zusammenarbeit zahlreicher Fachgesellschaften inklusive der ÖDG publiziert [21]. Besonders bemerkenswert ist, dass bereits eine gezielte Kurzintervention von 1–2 min eine Erfolgsquote von 2–5 % aufweist und somit aufgrund des geringen Zeitaufwandes als sehr effektiv zu betrachten ist. Eine kostenfreie Unterstützung wird österreichweit vom Rauchfrei-Telefon (www.rauchfrei.at) angeboten.

### Pharmakotherapie

Nikotinersatztherapie erhöht den Erfolg einer Raucherentwöhnung im Schnitt um 50–70 % [22]. Vareniclin erhöht die Erfolgswahrscheinlichkeit um das Doppelte bis Dreifache (verglichen mit Placebo) und ist im Schnitt Bupropion und Nikotinersatz überlegen (Absolutzahlen für kontinuierliche Abstinenzquoten nach 24 Wochen für Vareniclin, Bupropion, Nikotinersatztherapie, Placebo: 21,8 %, 16,7 %, 15,7 %, 9,4 %) [23].

Eine neue Therapieoption ist Cytisin, ein natürlicher α4β2-Nikotinrezeptor-Partialagonist und hat sich als effektive und kostengünstigere Alternative etabliert. Die Behandlungsdauer beträgt nur 25 Tage (im Gegensatz zu mehreren Monaten bei anderen Medikamenten), was die Adhärenz erhöht [24, 25]. Cytisin zeigt höhere Erfolgsraten als Bupropion und Nikotinersatztherapie, ist aber Vareniclin unterlegen [26]. Eventuelle Nebenwirkungen sind zu beachten. Vareniclin wurde hinsichtlich Wirkung und Sicherheitsprofil bei Menschen mit und ohne Diabetes verglichen und zeigte vergleichbare Erfolgsquoten und ein vergleichbares günstiges Nebenwirkungsprofil [27].

Eine wichtige neue Entwicklung in der Raucherentwöhnung ist der Einsatz von GLP-1-Rezeptoragonisten (GLP-1-RAs) wie Semaglutid und Dulaglutid als adjuvante Therapie [28]. Diese zeigen Effekte sowohl für die Raucherentwöhnung als auch für die Gewichtskontrolle nach dem Rauch- und Nikotinstopp, so reduzierte Dulaglutid substanzielle Gewichtszunahme nach dem Rauchstopp [29]. Ein möglicher Mechanismus ist, dass GLP-1-Rezeptoren in belohnungsrelevanten Hirnregionen (mesolimbisches Dopaminsystem) exprimiert sind und nikotininduzierte Belohnungseffekte modulieren [28]. GLP-1-RAs sollten somit bei rauchenden Personen mit Diabetes und Sorge vor einer Gewichtszunahme als adjuvante Therapie zur konventionellen Raucherentwöhnung (medikamentöse Therapie + Verhaltenstherapie) erwogen werden.

Ziel ist immer ein kompletter Rauch- und Nikotinstopp, auch wenn dies oft nur in Schritten zu erreichen ist. Nicht-tägliches Rauchen („Gelegenheitsrauchen“) reduziert die bei rauchenden Personen um den Faktor 2,32 erhöhte Gesamtmortalität nur auf das 1,93-Fache [30]. Weiters ist besonders in schwierigeren Lebensphasen eine Rückkehr ins ursprüngliche Rauchverhalten leichter möglich.

### Alternative Nikotinprodukte: erhitzte Tabakprodukte, E-Zigaretten und rauchfreie Nikotinprodukte

#### Erhitzte Tabakprodukte (HTPs)

Erhitzte Tabakprodukte (HTPs) sind Geräte, die echten Tabak bei niedrigeren Temperaturen (240–350 °C) erhitzen, ohne ihn zu verbrennen. Dabei wird behauptet, weniger schädliche Substanzen als bei herkömmlichen Zigaretten zu erzeugen. Da die Tabakindustrie von sinkenden Verkaufszahlen konventioneller Zigaretten betroffen ist, werden HTPs zunehmend als weniger schädliche Alternative vermarktet [31]. Hierbei wird allerdings mit konventionellen Zigaretten verglichen und daraus auf „gesündere“ Eigenschaften geschlossen [32]. In Wirklichkeit ist auch erhitzter Tabak im Vergleich mit Nichtrauchen mit Krankheit und Tod assoziiert [33].

Aktuelle Kohortenstudien mit 183.870 Teilnehmern zeigen deutlich höhere Risiken als bisher angenommen. HTPs erhöhen das Risiko für ein metabolisches Syndrom um 68 % (HR 1,68; 95 % CI: 1,25–2,26) im Vergleich zu Nie-Nutzern. Bei ausschließlichen HTP-Nutzern (ohne konventionelle Zigaretten) verdoppelt sich das Risiko bei Nutzung ≥ 3 Jahre (HR 2,17; 95 % CI: 1,31–3,62). Bei Nutzung von > 16 HTPs/Tag steigt das Risiko für metabolisches Syndrom um 33 % (HR 1,33; 95 % CI: 1,18–1,49). Bemerkenswert ist, dass das Risiko sogar höher scheint als bei konventionellen Zigaretten [34].

#### E-Zigaretten (elektronische Zigaretten)

E‑Zigaretten verdampfen aromatisierte Flüssigkeiten (sog. „Liquids“) mit oder ohne Nikotinzusatz. Die Zusammensetzung und die Inhaltsstoffe dieser Trägerflüssigkeiten sind seit 2014 durch die Tabakprodukterichtlinie 2014/EU (TPD II) [35] geregelt. Die Hauptinhaltstoffe sind Vernebelungsmittel (Propylenglykol und Glycerin), Chemikalienzusätze, pharmakologische Wirkstoffe, verschiedene Duft- und Aromastoffe (z. B. Menthol, Linalool) und Verunreinigungen. Eine effektive Kontrolle aller Substanzen und ihrer Wirkung auf den menschlichen Organismus ist fast unmöglich [36].

E‑Zigaretten sind seit 2014 unter Jugendlichen in den USA das am häufigsten verwendete Nikotinprodukt [37]. Vor allem bei Jugendlichen steigt mit dem Konsum von E‑Zigaretten auch das Risiko, mit dem Rauchen herkömmlicher Zigaretten zu beginnen, deutlich [38].

E‑Zigaretten gelten oft fälschlicherweise als sichere Alternative zum Rauchen, insbesondere unter schwangeren Frauen [39]. Die Nikotinkonzentration bei E‑Zigaretten ist variabel, das kinetische Profil ähnelt dem der herkömmlichen Zigarette [40], somit ist die Verwendung von E‑Zigaretten, vor allem in der Schwangerschaft, als besorgniserregend einzustufen [41].

Der mögliche Zusammenhang zwischen E‑Zigaretten und der Entwicklung eines metabolischen Syndroms bzw. Pathomechanismen im Glukosestoffwechsel sind noch nicht vollständig geklärt. Im Tierversuch gibt es Hinweise, dass das Rauchen von E‑Zigaretten, mit oder ohne Nikotin, vergleichbare Auswirkungen auf Gewicht und Glykämie hat wie das Rauchen herkömmlicher Zigaretten [42]. Eine koreanische Studie zeigte, dass der Dual Use von E‑Zigaretten und herkömmlichen Zigaretten sowie das Passivrauchen das Prädiabetesrisiko erhöhen. Im Vergleich mit Nichtrauchern war das Risiko für Prädiabetes mit einem 1,57-fachen, bei alleinigem Konsum von herkömmlichen Zigaretten mit einem 1,27-fachen Anstieg verbunden. Auch jene, die in der Vergangenheit herkömmliche sowie E‑Zigaretten konsumierten, hatten ein erhöhtes Risiko für Prädiabetes (OR 1,54; 95 % CI: 1,04–2,13) [43]. Eine in den USA durchgeführte, repräsentative Umfrage unter 600.046 Erwachsenen gab Hinweis auf einen statistisch nachweisbaren Zusammenhang zwischen dem Konsum von E‑Zigaretten und Prädiabetes [44]. Die Entwicklung einer viszeralen Adipositas sowie ein Anstieg der Triglyzeride wurden beobachtet [45].

#### Dual Use – höchstes Diabetesrisiko bei kombinierter Nutzung

Eine besonders besorgniserregende neue Erkenntnis ist das erhöhte Risiko bei sog. „Dual Use“ – der gleichzeitigen Nutzung von E‑Zigaretten und konventionellen Zigaretten. Eine Studie von 2024 mit 320 Teilnehmern zeigt, dass Dual User die schlechteste glykämische Kontrolle aufweisen. Dual User mit Diabetes hatten HbA_1c_-Werte von 11,84 ± 1,47 % vs. 10,95 ± 1,45 % bei reinen Zigarettenrauchern. Dual User zeigen die höchsten HOMA-IR-Werte (8,15 ± 0,89) im Vergleich zu reinen Zigarettenrauchern (7,03 ± 0,81) oder Kontrollen (2,02 ± 0,94). Das erhöhte Risiko für Dual Use ist besonders ausgeprägt bei männlichen, inaktiven und adipösen Personen [46].

#### Rauchfreie Nikotinprodukte (Nikotinbeutel)

Rauchfreie Nikotinprodukte wie Nikotinbeutel stellen eine weitere Kategorie nikotinhaltiger Produkte dar, die weder geraucht noch verdampft werden. Diese Produkte werden zwischen Lippe und Zahnfleisch platziert und geben Nikotin über die Mundschleimhaut ab. Nikotinbeutel enthalten synthetisches Nikotin, Pflanzenfasern, Aromen und Süßstoffe, aber keinen Tabak.

Auch hier zeigt die aktuelle Evidenz alarmierende Zusammenhänge mit Diabetes. So ergab eine Metaanalyse von 5 Kohortenstudien ein erhöhtes Typ-2-Diabetes-Risiko bei hohem Konsum von Nikotinbeuteln (≥ 7 Dosen/Woche) [47]. Eine schwedische Kohortenstudie zeigte bei jungen Erwachsenen, dass exklusiver Konsum von Nikotinbeuteln mit höherem BMI und Taillenumfang assoziiert war [48]. Orale Gesundheitsrisiken umfassen Mund- und Zahnfleischirritationen, Parodontose, sowie entzündliche Reaktionen der Mundschleimhaut mit erhöhten Entzündungsmarkern (TNF‑α, IL‑6, IL-8) [49].

Die schnelle Nikotinabsorption über die Mundschleimhaut kann zu sehr hohen Nikotinspiegeln führen – viel höher als bei regulärer Nikotinersatztherapie. Dies macht diese Produkte hochgradig abhängigkeitsfördernd [50]. Wegen der multiplen Gesundheitsrisiken und des hohen Abhängigkeitspotenzials werden Nikotinbeutel somit nicht zur Entwöhnung von Tabakprodukten empfohlen.

#### Zusammenfassung der Risiken alternativer Nikotinprodukte

Weder erhitzte Tabakprodukte noch E‑Zigaretten noch rauchfreie Nikotinprodukte sind sichere Alternativen zum Rauchen. Alle Produktkategorien sind mit erhöhten Gesundheitsrisiken verbunden, insbesondere im Zusammenhang mit dem metabolischen Syndrom und Diabetes. Eine aktuelle Zusammenfassung von erhitzten Tabakprodukten findet sich in einer Empfehlung der „Initiative Ärzte gegen Raucherschäden“ (www.aerzteinitiative.at), für die auch die ÖDG mitverantwortlich zeichnet [33].

## 2. Psychische und sozioökonomische Aspekte

Wie für die Entstehung des Typ-2-Diabetes selbst wird zunehmend die Bedeutung sozioökonomischer und psychischer Faktoren auch im Zusammenhang mit Diabetes und Rauchverhalten deutlich. So zeigte die Young Finns Study [51], in welcher Menschen über einen Zeitraum von 31 Jahren von der Kindheit bis ins mittlere Erwachsenenalter prospektiv nachverfolgt wurden, dass jene Personen, die in einer sozioökonomisch stark benachteiligten Nachbarschaft aufwuchsen, ein um fast das 4‑Fache erhöhtes relatives Risiko (RR 3,71; *p* = 0,0008) hatten, einen Diabetes zu entwickeln, als jene, die in einer Nachbarschaft mit der geringsten sozioökonomischen Benachteiligung lebten. Bereits ab dem 6. Lebensjahr aß die Gruppe mit hoher sozioökonomischer Benachteiligung weniger Obst und Gemüse (*p* < 0,0001), übte ab dem 12. Lebensjahr weniger körperliche Aktivität aus (*p* = 0,007) und rauchte ebenfalls ab dem 12. Lebensjahr häufiger täglich (*p* < 0,0001).

In der Literatur gibt es Hinweise dafür, dass es Patient:innen mit Diabetes besonders schwerfällt, das Rauchen aufzugeben [52]. Als Gründe dafür werden verschiedene Ursachen angeboten: Patient:innen mit Typ-2-Diabetes scheinen Nikotin schneller zu metabolisieren [53]. Personen mit schnellerem Nikotinmetabolismus rauchen mehr Zigaretten [54] und haben eine geringere Wahrscheinlichkeit, das Rauchen aufzugeben, als Personen mit langsamerem Nikotinabbau [55]. Darüber hinaus legen Daten aus dem Tierversuch nahe, dass erhöhte Glukosespiegel selbst die Belohnungseffekte von Nikotin im Gehirn steigern [56]. Diesem belohnungssteigernden Effekt konnte – ebenfalls im Tiermodell – durch Blutzuckersenkung mit Insulin oder Dapagliflozin entgegengewirkt werden [57].

Daten aus klinischen Untersuchungen sprechen für das bei Diabetes häufige Auftreten einer komorbiden Depression [58] oder die besonders hohe Belastung des Rauch- und Nikotinstopps bei einer ohnehin im (Selbst‑)Management aufwendigen chronischen Erkrankung [59] als mögliche Ursache des erschwerten Verzichts auf Nikotin. Ein großer nationaler Diabetes-Audit in Australien [60] zeigte, dass fast ein Drittel der in Diabeteszentren behandelten Patient:innen mit Typ-2-Diabetes von wahrscheinlicher Depression und Diabetes-Distress betroffen waren, ein erheblicher Teil jedoch unbehandelt blieb. Patient:innen mit Depression oder Diabetes-Distress hatten eine geringe Wahrscheinlichkeit, die Empfehlungen zu Rauch- und Nikotinstopp, Diät, körperlicher Aktivität und Glukosemonitoring umzusetzen. Auch in einer kanadischen Untersuchung wurde bei Menschen mit Typ-2-Diabetes eine starke Assoziation zwischen Depression und Rauchen gefunden [61]. Übereinstimmend mit den australischen Daten war auch in diesem Kollektiv die Prävalenz an Depression unter jenen, die erfolglos versucht hatten, das Rauchen aufzugeben, höher als bei jenen, die erfolgreich aufgehört hatten.

### Klinische Empfehlungen

Wegen der enormen Bedeutung von Rauchen und Nikotinkonsum für Genese und Prognose des Diabetes und der nachgewiesenen starken Risikoreduktion durch einen vollständigen Rauch- und Nikotinstopp sollte jeder Betroffene mit Diabetes regelmäßig (mindestens 1‑mal jährlich) nach einem eventuellen Tabak- und Nikotinkonsum befragt und über das erhöhte Diabetesrisiko und Spätschäden informiert werden. Bei Tabak- oder Nikotinabhängigkeit sollte eine weiterführende Diagnostik durchgeführt werden. Bei chronischem Tabak- oder Nikotinrauchen ist ein vollständiger Rauch- und Nikotinstopp dringlich zu empfehlen.

Jede nikotinabhängige Person mit Diabetes sollte ein strukturiertes Therapieangebot, bei höherem Abhängigkeitsgrad mit medikamentöser Unterstützung, zur Behandlung seiner Abhängigkeit bekommen. Dem Zusammenhang zwischen sozioökonomischem Status, psychischen Begleitfaktoren, Diabetes und Rauchverhalten sollte von sozial- und gesundheitspolitischer Seite Beachtung geschenkt werden.

## 3. Alkohol

Prinzipiell wurden geringe Mengen an täglicher Alkoholzufuhr für Patient:innen mit Diabetes mellitus als nicht gesundheitsschädigend, sondern hinsichtlich Erkrankungen des kardiovaskulären Systems als eher protektiv gesehen [62]. Mögliche mechanistische Erklärungen dafür gehen von einer gering senkenden Wirkung auf die LDL-Cholesterin-Konzentration, signifikanten Erhöhung des HDL-Cholesterins und Hemmung von Entzündungsmediatoren wie Interleukin‑6 aus [63]. Diese Annahme schlägt sich auch in aktuellen Leitlinien zur Ernährung bei Diabetes mellitus nieder. So ist in den aktuellen Ernährungsempfehlungen der Amerikanischen Diabetes Gesellschaft (ADA) für Frauen ein alkoholischer „Drink“ (max. 20 g Alkohol, entspricht ca. 1/8 Wein, einem Glas Bier zu 0,3 l oder einer Einfachdosis einer Spirituose) pro Tag freigegeben, für Männer maximal 2, wobei auf das erhöhte Hypoglykämierisiko unter Alkoholeinfluss besonders hingewiesen wird [64].

Menschen mit Diabetes mellitus, die bisher Alkohol-abstinent waren, sollen laut ADA keinesfalls beginnen, Alkohol zu konsumieren, auch wenn manche Studien von einer verringerten Diabetesneumanifestation bei Personen, die 5 bis 10 „Drinks“ pro Woche zu sich nehmen, im Vergleich zu abstinenten Individuen berichten [65].

Neuere Forschung zum Thema Alkoholkonsum geht aber eher von einem „negativen Selektionsbias“ hinsichtlich des vorbestehenden Gesundheitszustands und/oder des sozialen Status bei „völlig abstinenten“ Personen mit keinerlei Alkoholkonsum aus, zudem nimmt man an, dass in epidemiologischen Studien falsch niedrige Angaben zum Alkoholkonsum gemacht werden. In diesem Sinne werden gesundheitsfördernde Effekte von Alkohol prinzipiell verneint und die medizinischen Folgeschäden in den Mittelpunkt der Diskussion gestellt [66, 67]. In einer großen internationalen epidemiologischen Studie (GBD 2016 Alcohol Collaborators) wurde Alkoholkonsum als einer der führenden Risikofaktoren für globale Krankheitslast bezeichnet und für beträchtlichen Verlust an Gesundheit, gemessen an Mortalitätsüberschuss und Verlust an behinderungsfreien Jahren, verantwortlich gemacht [68]. In den krankheitsbezogenen Detailanalysen wurde bei mäßigem Alkoholkonsum zwar eine Reduktion des relativen Risikos für Diabetes mellitus und ischämische Herzkrankheit bei Männern und Frauen beschrieben, global bezogen auf alle alkoholassoziierten Gesundheitsstörungen (dabei vorrangig Krebs und Infektionserkrankungen) kam es aber zu einem stetig ab Null ansteigenden relativen Risiko für Verlust an behinderungsfreien Lebensjahren mit jedem täglich getrunkenen Schluck Alkohol („Drink“). Dabei dürfte vor allem die dosisbezogene Steigerung der Krebshäufigkeit einen wesentlichen Anteil haben, da australische Forscher jüngst errechnet haben, dass eine Reduktion des jährlichen Konsums von reinem Alkohol um drei Liter pro Kopf (Schätzung für Deutschland: durchschnittlich 11 l Reinalkohol/Jahr und Bürger) die Todesfälle durch Krebs in den nächsten 20 Jahren um 12 % senken würde [69]. Als Mediator dafür wird das Zwischenprodukt des Alkoholabbaus Acetaldehyd angeschuldigt, welches laut aktueller Grundlagenforschung dosisabhängig die DNA schädigen dürfte [70].

In einem aktuellen Review wird ausgeführt, dass sich die oberste Gesundheitsbehörde der USA für eine Kennzeichnung („labeling“) alkoholhaltiger Produkte ausspricht, welche auf das erhöhte Krebsrisiko durch Alkoholkonsum hinweist. Ähnliche Initiativen gibt es in Irland, Südkorea, Kanada, Norwegen und Thailand. Laut der World Health Organisation (WHO) wurden 2020 global 740.000 Krebsfälle (also 4,1 % der Gesamtzahl) durch Alkohol verursacht, bei Männern deutlich häufiger (76,7 %) als bei Frauen. In den USA wird Alkohol (auch leichter und moderater Konsum) nach Zigarettenrauchen und Übergewicht als dritt häufigster modifizierbarer Auslöser von Krebserkrankungen (verantwortlich für 5,4 % der Fälle) gesehen. Aus Sicht der WHO gibt es daher keine „sichere“ Untergrenze für die Alkoholzufuhr [71].

Eine der häufigsten durch Alkoholkonsum ausgelösten onkologischen Erkrankungen ist das hepatozelluläre Karzinom als Folge der durch Alkohol verursachten Leberfibrose bis hin zur Leberzirrhose. Beide können aber auch durch eine fortschreitende Fettlebererkrankung (MASLD, MASH) verursacht sein, die bei Menschen mit Adipositas und Typ-2-Diabetes gehäuft auftritt. Bei zusätzlichem regelmäßigem Alkoholkonsum wird von MetALD („Metabolic dysfunction and Alcohol-related Liver Disease“) gesprochen, die in Österreich häufig zu erheben ist und bei der Alkoholkarenz von zentraler prognostischer und therapeutischer Bedeutung ist [72].

Eine rezente Studie, welche 371.463 Personen mit vorliegender genetischer Analyse einschloss (UK Biobank), zeigte, dass leichter bis moderater Alkoholkonsum mit gesünderem Lebensstil assoziiert war, der mitverantwortlich für die beobachtete kardioprotektive epidemiologische Assoziation mit moderatem Alkoholkonsum sein dürfte. Eine non-lineare „Mendelian-Randomization“-Analyse mit genetisch vorhersagbarem Alkoholkonsum ergab eine minimale Erhöhung des kardiovaskulären Risikos durch leichten Alkoholkonsum, aber eine exponentielle Zunahme von klinischer und subklinischer kardiovaskulärer Erkrankung bei höherer Alkoholzufuhr [73].

Neueste epidemiologische Daten zeigen, dass die altersadjustierten Raten an karzinomassoziierten DALYs („disability adjusted life years“) in einer Analyse des „Global Burden of Diseases“ in folgender Reihenfolge der genannten Faktoren verursacht werden: 1. Rauchen, 2. Alkoholgebrauch, 3. hoher Body-Mass-Index, 5. erhöhte Nüchternglukose; Passivrauchen erreichte die 10. Stelle [74].

Eine aktuelle koreanische Studie bei über 4,5 Mio. Personen ohne spezifische Diagnosedifferenzierung zeigte bei Analyse von Fragebogendaten zu Lebensstilverhalten, dass bei Zunahme des Alkoholkonsums über die Zeit das Risiko für Alkohol-assoziierte Krebsarten, aber auch für alle erhobenen Krebsarten signifikant steigt, während bei Alkoholabstinenz oder Verringerung des Alkoholkonsums diese fällt, weswegen Letzteres im Sinne der Krebsprävention anzustreben und zu unterstützen ist [75].

Für Personen mit Diabetes mellitus zeigt eine retrospektive Studie in einem großen finnischen Kollektiv, dass das Risiko für Alkohol-assoziierte Todesfälle für Individuen mit Diabetes unter oraler Therapie um den Faktor 1,71 höher liegt als bei Personen ohne Diabetes, für Patient:innen unter Insulintherapie sogar um das fast 7‑Fache (RR 6,92) [76].

Möglicherweise ist die höhere „Toxizität“ von Alkohol bei Patienten mit Diabetes mellitus durch das häufig gleichzeitige Vorliegen von metabolisch bedingten Hepatopathien, ausgeprägten Fettstoffwechselstörungen (z. B. exzessiver Hypertriglyzeridämie) und Erkrankungen des exokrinen Pankreas erklärbar. Vor allem bei Lebererkrankungen ist chronischer Alkoholkonsum – in welcher Menge auch immer – kritisch zu sehen [77].

In diesem Zusammenhang ist auch zu ergänzen, dass Personen mit regelmäßigem Alkoholkonsum in einem Programm zur chronischen Gewichtsreduktion (Look AHEAD Study) deutlich weniger Gewicht verloren als jene mit Alkoholabstinenz [78].

In einer kürzlich publizierten Untersuchung wurde das Alkoholkonsumverhalten vor bzw. nach der Diagnose eines Typ-2-Diabetes mit dem Auftreten von neu auftretendem Vorhofflimmern in Beziehung gesetzt: Personen, die vor der Diagnose des Diabetes mehr als 40 g Alkohol/Tag konsumierten oder mehr als 3‑mal pro Woche tranken, hatten im Vergleich zu Nicht-Trinkern ein signifikant erhöhtes Risiko für das Auftreten eines Vorhofflimmerns (adjustierte HR = 1,22; 95 % KI 1,06–1,41 bzw. 1,13; 95 % KI 1,03–1,25). Personen, welche weniger als 40 g/Tag oder weniger als 3‑mal in der Woche tranken, hatten kein erhöhtes Risiko [79]. Bezüglich des Trinkverhaltens nach der Diagnose des Typ-2-Diabetes zeigte sich, dass im Vergleich zu jenen, die weiterhin ≥ 20 g/Tag Alkohol konsumierten, jene, die ihren Konsum auf < 20 g/Tag reduzierten, ein um 19 % (HR 0,81; 95 % CI 0,68–0,97) geringeres und dauerhafte Nicht-Trinker ein um 20 % (aHR = 0,80; 95 % CI 0,69–0,92) geringeres Risiko aufwiesen. In dieser Studie konnte erstmals gezeigt werden, dass starker und häufiger Alkoholkonsum bei Patient:innen mit Diabetes mit einem erhöhten Risiko für Vorhofflimmern assoziiert ist und dass bei Personen, die den Konsum deutlich reduzieren, dieses Risiko auf das Niveau von Nicht-Trinkern sinkt. Auch wenn die Generalisierbarkeit der Ergebnisse aufgrund methodischer Kritikpunkte offenbleibt [80], entsprechen diese Resultate der in einem Kollektiv mit sehr geringem Anteil an Patient:innen mit Diabetes beobachteten positiven Auswirkung der Alkoholabstinenz auf das Auftreten des Vorhofflimmerns [81].

Aus diabetologischer Sicht ist bei Vorliegen einer Alkoholerkrankung hinsichtlich neuer Therapieoptionen anzumerken, dass GLP-1-Rezeptoragonisten – konkret Semaglutide subkutan in einer Dosis bis zu 1,0 mg wöchentlich – das „Craving“ und andere „drinking outcomes“ in einer Phase-2-Studie über 9 Wochen bei alkoholkranken Personen vs. Placebo signifikant verbessern konnte [82].

Interessant erscheint auch beim Trend zu alkoholfreien Getränken, dass alkoholfreie Biere als Geschmacksträger im Allgemeinen statt Alkohol mehr Kohlenhydrate enthalten, kohlenhydratreduzierte („low-carb“) Biere durch verstärkte Vergärung einen eher höheren Alkoholgehalt aufweisen.

Die Entscheidung, ob nun ein „Drink“ pro Tag bei Menschen mit Diabetes mellitus ärztlich erlaubt oder verboten wird, ist also individuell zu treffen. Eine ärztliche „Empfehlung“ dafür kann in Kenntnis der rezenten Literatur aber nicht ausgesprochen werden.

## 4. Infektionen und Impfungen

### Überblick

Menschen mit Diabetes mellitus zeigen im Vergleich zur Gesamtbevölkerung eine deutlich erhöhte Anfälligkeit für Infektionserkrankungen. Besonders betroffen sind Infektionen der Atemwege, wie Influenza und Pneumokokken-Pneumonie, aber auch Harnwegsinfektionen, Haut- und Weichteilinfektionen sowie invasive bakterielle Infektionen (z. B. Pneumokokken-Sepsis und -Meningitis) treten gehäuft auf [83–85]. Das Infektionsrisiko ist sowohl bei Diabetes Typ 1 als auch bei Diabetes Typ 2 erhöht und steigt weiter mit zunehmender Krankheitsdauer, schlechter glykämischer Kontrolle, dem Vorhandensein diabetischer Komplikationen und mit dem Lebensalter [83, 84]. So ist z. B. die Häufigkeit, an einer Pneumokokken-Pneumonie zu erkranken, bei älteren Personen mit Diabetes fast 3‑mal so hoch wie in gleichaltrigen gesunden Kontrollen [83, 86]. Für invasive Pneumokokken-Erkrankungen wurde eine 2,3- bis 4,6-fach erhöhte Inzidenz bei Menschen mit Diabetes nachgewiesen [83, 86]. Auch die Mortalität als Folge einer bakteriellen Lungenentzündung ist deutlich erhöht: Die Wahrscheinlichkeit, innerhalb von 90 Tagen an einer bakteriellen Pneumonie zu versterben, ist 2‑ bis 3‑fach höher als bei Menschen ohne Diabetes [85].

Infektionen im Allgemeinen, auch jene ohne initiale Hospitalisierung, erhöhen zudem das Risiko für kardiovaskuläre Ereignisse sowie neurodegenerative Erkrankungen wie Demenz [90–93]. Der Zusammenhang zwischen Infektionen und späterem Auftreten einer Demenz ist bei Diabetes stärker ausgeprägt [90]. Impfungen zum Schutz vor Influenza oder Herpes Zoster reduzieren nachweislich das Demenzrisiko [92, 93]. Des Weiteren können Impfungen das Risiko für schwerwiegende kardiovaskuläre Ereignisse senken, besonders bei Risikopatient:innen. Die European Society for Cardiology (ESC) empfiehlt Impfungen gegen Influenza, SARS-CoV‑2, Pneumokokken und RSV im Rahmen der kardiovaskulären Prävention [86, 94]. Kinder und Erwachsene mit Diabetes sollten gemäß den Empfehlungen des Österreichischen Impfplans geimpft werden [87]; aufgrund des erhöhten Risikos wird teils zu einer früheren Impfung geraten. Auch die American Diabetes Association (ADA) hat 2024 bereits Impfempfehlungen in ihren Leitlinien integriert [88].

### Potenzielle Mechanismen

Hinter dieser gesteigerten Infektionsanfälligkeit stehen verschiedene pathophysiologische Mechanismen. Die Basis bildet eine gestörte Immunabwehr auf mehreren Ebenen. Chronisch erhöhte Blutzuckerwerte beeinträchtigen die Funktion neutrophiler Granulozyten und Makrophagen – ihre Chemotaxis, Phagozytose und bakterizide Wirkung nehmen ab [97–99]. Auch die spezifische Immunität ist geschwächt: T‑Zellen und humorale Immunität können in ihrer Funktion gestört sein, was sich unter anderem in einer verminderten Impfantwort bei schlechter glykämischer Kontrolle äußert [96, 99]. Zusätzlich sind die lokalen Abwehrmechanismen gestört, etwa die mukoziliäre Clearance der Atemwege, was die Elimination inhalierter Keime beeinträchtigt [101]. Systemische Komorbiditäten wie Mikro- und Makroangiopathien, Polyneuropathie, chronisch entzündlicher Zustand und eingeschränkte Organfunktionen (z. B. Niereninsuffizienz) fördern zusätzlich Infektionen und erschweren die Heilung [98, 102, 103]. Auch mit der Anzahl der Begleiterkrankungen steigt das Risiko für schwere Pneumonie- und Sepsisverläufe [83]. Weiters können akute Infektionen ebenfalls zu einer ausgeprägten systemischen Entzündungsreaktion des Gefäßsystems mit Aktivierung des Endothels, prothrombotischer Verschiebung und transienter Instabilisierung vulnerabler atherosklerotischer Plaques führen [98, 99]. Für das Gehirn werden unter anderem durch Infektionen getriggerte neuroinflammatorische Prozesse mit Mikrogliaaktivierung und Barrieredysfunktion diskutiert, die das Demenzrisiko langfristig erhöhen können [90, 92, 93, 103].

### Klinik oft different: atypische Verläufe von Infektionen bei Diabetes

Infektionen treten bei Menschen mit Diabetes häufig mit untypischen, abgeschwächten oder unspezifischen Symptomen auf. Klassische Zeichen wie Fieber und ein akuter Krankheitsbeginn können fehlen oder nur gering ausgeprägt sein, insbesondere bei älteren Menschen oder bei multimorbiden Patient:innen [104]. Das erschwert oft die frühzeitige Erkennung schwerer Infektionsverläufe in dieser Patient:innengruppe. Auf der anderen Seite sind die metabolischen Folgen häufig umso dramatischer: Infektionen können rasch eine Dekompensation der Stoffwechsellage auslösen, was in einer diabetischen Ketoazidose oder einem hyperosmolaren Koma münden kann [107–109]. Zudem kommt es häufiger zu kardiovaskulären Komplikationen wie Myokardinfarkt, Schlaganfall oder Verschlechterung einer bestehenden Herzinsuffizienz. So bewirkt eine Infektion mit Influenzaviren bei Diabetesbetroffenen ein deutlich erhöhtes Risiko für Hospitalisierung, Intensivpflichtigkeit und Mortalität; das Risiko für kardiovaskuläre Ereignisse unmittelbar nach der Infektion ist bei Menschen mit Diabetes besonders hoch [111–113]. Diese atypischen Präsentationen unterstreichen die Bedeutung präventiver Maßnahmen wie Impfungen, Schulungen und im Fall einer Infektion engmaschiger klinischer Überwachung [86, 87].

### Impfungen bei Menschen mit Diabetes: klinische Relevanz und Beispiele

#### Influenza

Menschen mit Diabetes erkranken häufiger und schwerer an Influenza, mit höherem Risiko für Hospitalisierung, Komplikationen und Tod. Die Influenza-Impfung ist bei Diabetes wirksam [96, 113], reduziert die Zahl der Hospitalisierungen [113] und senkt bei Menschen mit Diabetes und Herz-Kreislauf-Erkrankungen das Risiko für kardiovaskuläre Ereignisse und Mortalität [111, 112]. Eine Impfung senkt das Risiko für schwerwiegende unerwünschte kardiovaskuläre Ereignisse um 34 % und bei kürzlich stattgehabtem ACS um 45 % [111, 112]. Daher empfiehlt die ESC eine Impfung für alle Patient:innen mit ACS mit höchster Empfehlungsstärke (Klasse I, Evidenzgrad A) [86, 94].

Laut Österreichischem Impfplan ist die jährliche Influenza-Impfung besonders für Risikogruppen wie Menschen mit Diabetes sowie deren Kontaktpersonen empfohlen. Für Personen ab dem vollendeten 60. Lebensjahr, und noch nachdrücklicher ab dem vollendeten 65. Lebensjahr, wird ein adjuvantierter oder Hochdosisimpfstoff empfohlen, welche in dieser Altersgruppe höhere Antikörperspiegel induzieren [87]. Es findet sich ein ähnliches Impfansprechen bei Menschen mit und ohne Diabetes [96].

#### COVID-19

Menschen mit Diabetes haben ein erhöhtes Risiko für einen schweren COVID-19-Erkrankungsverlauf einschließlich erhöhter Hospitalisierungsraten, Komplikationen und Mortalität [97, 99]. Ähnliche Ergebnisse berichtet die International Diabetes Federation (IDF): So ist bei Patient:innen mit deutlich erhöhtem HbA_1c_ das Mortalitätsrisiko etwa um das 3‑Fache erhöht, was den Einfluss einer unzureichenden Blutzuckerkontrolle auf den Krankheitsverlauf unterstreicht [95]. Bei Personen mit Diabetes wird häufiger ein „inflammatory storm“ und hyperkoagulatorischer Zustand beobachtet. Des Weiteren kann SARS-CoV‑2 den Glukosestoffwechsel stören und die glykämische Kontrolle verschlechtern, was wiederum den Erkrankungsverlauf erschweren kann. Der Schweregrad des Diabetes korreliert dabei direkt mit der Prognose; insbesondere bei Vorliegen diabetischer Komplikationen ist die Sterblichkeit deutlich erhöht [97, 99]. Zudem zeigte eine Metaanalyse mit fast 40 Mio. Personen ein um 62 % erhöhtes Risiko für eine Diabetesdiagnose nach SARS-CoV-2-Infektion sowohl für Typ-1- als auch Typ-2-Diabetes, besonders nach schwerem Verlauf [100].

Die Immunantwort auf COVID-19-Impfstoffe ist bei guter glykämischer Kontrolle vergleichbar mit der von Menschen ohne Diabetes, kann jedoch bei schlechter glykämischer Kontrolle aufgrund einer eingeschränkten neutralisierenden Antikörperbildung und CD4-T-Zell-Antwort beeinträchtigt sein [98].

Eine COVID-19-Auffrischungsimpfung wird besonders Menschen mit Diabetes empfohlen, um das Risiko eines schweren Verlaufs zu reduzieren; idealerweise im Herbst [87]. Die Impfung reduziert nachweislich das Risiko für schwere Verläufe und Todesfälle und führt bei Durchbruchsinfektion dennoch zu milderen Verläufen [87, 95]. Zudem verringert sie das Risiko für Long-COVID, das bei Diabetes erhöht sein kann [101].

#### Pneumokokken

Menschen mit Diabetes haben ein erhöhtes Risiko für schwere Pneumokokken-Erkrankungen wie Pneumonie und invasive Erkrankungen, intensivmedizinische Behandlungen und Mortalität [83–85, 102]. Das Risiko invasiver Pneumokokken-Erkrankungen ist bei Erwachsenen mit Diabetes 2‑ bis 3‑fach höher als in der Allgemeinbevölkerung, wobei das Risiko mit dem Alter weiter steigt [83].

Akute Pneumokokken-Erkrankungen können durch Toxine und Entzündungsreaktionen zu Organschäden führen, die bestehende Erkrankungen verschlechtern sowie neue nach sich ziehen [85]. Die tatsächliche Langzeitbelastung wird dabei oft unterschätzt.

Die Pneumokokken-Impfung reduziert bei Menschen mit Diabetes signifikant Pneumonien und Hospitalisierungen [83]. Sie wird allgemein für Kinder bis zum vollendeten 2. Lebensjahr, gesunde Erwachsene ab dem vollendeten 60. Lebensjahr sowie Risikogruppen wie Diabetes empfohlen [87]. Die ADA empfiehlt Kindern mit Diabetes im Alter von 6 bis 18 Jahren eine Dosis (PPSV23), Erwachsenen mit Diabetes im Alter von 19 bis 64 Jahren eine Dosis (PCV20) [88]. Die Wirksamkeit scheint nach etwa 5 Jahren nachzulassen, dennoch wird ein Mindestabstand von 6 Jahren zwischen 2 Impfungen empfohlen, da kürzere Intervalle mit stärkeren Lokalreaktionen verbunden sind [87].

#### „Respiratory syncytical virus“ (RSV)

Personen mit Komorbiditäten, Immunschwäche und höherem Alter haben ein erhöhtes Risiko für schwere RSV-Infektionen. Das Risiko für eine RSV-bedingte Hospitalisierung ist bei Erwachsenen ab 45 Jahren mit Diabetes 2‑ bis 4‑fach erhöht [102]. RSV-Infektionen können nicht nur schwere Atemwegserkrankungen, sondern auch kardiovaskuläre Komplikationen verursachen. Beinahe ein Viertel der hospitalisierten Erwachsenen ab 50 Jahren erleidet ein akutes kardiales Ereignis [103]. Das Risiko ist bei vorbestehenden Herz-Kreislauf-Erkrankungen mehr als 3‑fach erhöht. Da viele Menschen mit Diabetes kardiovaskuläre Risikofaktoren oder Begleiterkrankungen haben, sind sie besonders anfällig für schwere RSV-Verläufe und sollten geimpft werden [86, 87].

Die Impfung ist ab dem vollendeten 60. Lebensjahr allgemein empfohlen, bei Risikogruppen wie Personen mit Diabetes bereits ab dem 18. Lebensjahr [87].

#### Hepatitis B

Menschen mit Diabetes haben ein erhöhtes Risiko für Hepatitis B [108], sodass eine Impfung empfohlen wird und je nach individuellem Risiko auch nach dem 60. Lebensjahr noch nachgeholt werden kann [87, 88]. Aufgrund einer häufig schwächeren Hepatitis-B-Impfantwort wird eine Titerkontrolle ab 6 Monaten nach der Grundimmunisierung empfohlen [108]. Bei ausreichendem Titer (≥ 100 mIE/ml) sollten bei andauernder Indikation Auffrischungsimpfungen alle 10 Jahre erfolgen [87].

#### Herpes Zoster

Menschen mit Diabetes haben laut einer rezenten Metaanalyse ein 38 % höheres Risiko für Herpes Zoster (HZ) und dessen Komplikationen wie bakterielle Superinfektion, Zoster ophthalmicus oder postherpetische Neuralgie sowie für eine Hospitalisierung. Höheres Alter, bestehende Herz-Kreislauf-Erkrankungen oder chronische Niereninsuffizienz sind weitere Risikofaktoren, die das HZ-Risiko erhöhen [104–106]. Innerhalb eines Jahres nach HZ ist das Risiko für Insult und Myokardinfarkt, besonders bei Diabetes, um das 1,3- bis 4‑Fache erhöht, da HZ entzündliche Prozesse in den Gefäßen begünstigt [106, 107].

Die HZ-Impfung wird allgemein ab 60 Jahren und für Personen mit erhöhtem Risiko wie Diabetes ab 18 Jahren empfohlen. Der adjuvantierte Herpes-Zoster-Subunit-Totimpfstoff zeigt eine hohe und lang persistierende Wirksamkeit über mindestens 10 Jahre hinsichtlich der Prävention von HZ und der postherpetischen Neuralgie [87]. Die Impfung senkt nachweislich das Demenzrisiko [92] und damit verbundene Gesundheitskosten [107].

#### Humane Papillomaviren (HPV)

Frauen mit Typ-1- und Typ-2-Diabetes haben ein erhöhtes Risiko für die meisten HPV-assoziierten Präkanzerosen und Karzinome, wobei die Krebsraten bei T2D tendenziell höher waren [110]. Auch Männer wiesen ein deutlich höheres Risiko auf, wobei dieses nur bei T2D nachweisbar war [109]. Daher sollten Screening und Impfung bei Diabetes priorisiert werden.

Die Impfung wird allen, frühestmöglich ab dem vollendeten 9. Lebensjahr, jedenfalls bis zum vollendeten 30. Lebensjahr empfohlen, danach optional je nach individuellem Risiko. Je früher die Impfung erfolgt, desto ausgeprägter ist ihr prophylaktischer Effekt [87].

Zusammenfassend ist somit zu sagen, dass Infektionen bei Personen mit Diabetes häufiger auftreten, schwerer und häufig atypisch verlaufen. Bei steigender Blutglukose ohne erkennbare andere Ursachen sollte immer auch an eine Infektion gedacht werden. Impfungen sind für Patient:innen mit Diabetes von noch größerer gesundheitlicher Relevanz als für Personen ohne Diabetes.
